# Current knowledge of COVID-19 and infection prevention and control strategies in healthcare settings: A global analysis

**DOI:** 10.1017/ice.2020.237

**Published:** 2020-05-15

**Authors:** M. Saiful Islam, Kazi M. Rahman, Yanni Sun, Mohammed O. Qureshi, Ikram Abdi, Abrar A. Chughtai, Holly Seale

**Affiliations:** 1 School of Public Health and Community Medicine, University of New South Wales, Sydney, Australia; 2 Program for Emerging Infections, Infectious Diseases Division, International Centre for Diarrhoeal Disease Research, Bangladesh (icddr,b), Dhaka, Bangladesh; 3 North Coast Public Health Unit, New South Wales Health, Lismore, New South Wales, Australia; 4 The University of Sydney, University Centre for Rural Health, Lismore, New South Wales, Australia; 5 Centre for Population Health, New South Wales Health, Sydney, Australia

## Abstract

**Objective:**

In the current absence of a vaccine for COVID-19, public health responses aim to break the chain of infection by focusing on the mode of transmission. We reviewed the current evidence on the transmission dynamics and on pathogenic and clinical features of COVID-19 to critically identify any gaps in the current infection prevention and control (IPC) guidelines.

**Methods:**

In this study, we reviewed global COVID-19 IPC guidelines by organizations such as the World Health Organization (WHO), the US Centers for Disease Control and Prevention (CDC), and the European Centre for Disease Prevention and Control (ECDC). Guidelines from 2 high-income countries (Australia and United Kingdom) and from 1 middle-income country (China) were also reviewed. We searched publications in English on ‘PubMed’ and Google Scholar. We extracted information related to COVID-19 transmission dynamics, clinical presentations, and exposures that may facilitate transmission. We then compared these findings with the recommended IPC measures.

**Results:**

Nosocomial transmission of SARS-CoV-2 in healthcare settings occurs through droplets, aerosols, and the oral–fecal or fecal–droplet route. However, the IPC guidelines fail to cover all transmission modes, and the recommendations also conflict with each other. Most guidelines recommend surgical masks for healthcare providers during routine care and N95 respirators for aerosol-generating procedures. However, recommendations regarding the type of face mask varied, and the CDC recommends cloth masks when surgical masks are unavailable.

**Conclusion:**

IPC strategies should consider all the possible routes of transmission and should target all patient care activities involving risk of person-to-person transmission. This review may assist international health agencies in updating their guidelines.

The global outbreak of coronavirus disease (COVID-19) is caused by the novel severe acute respiratory syndrome coronavirus 2 (SARS-CoV-2). During the last 20 years, 2 other coronavirus epidemics, SARS-CoV and Middle East respiratory syndrome (MERS)-CoV, have resulted in a considerable burden of cases across multiple countries.^1,2^ Outbreaks of newly emerging or remerging infectious diseases present a unique challenge and a threat to healthcare providers (HCPs) and other frontline responders due to limited understanding of the emerging threat and reliance on infection prevention and control (IPC) measures that may not consider all transmission dynamics of the emerging pathogens. Furthermore, HCP understanding and skills around the use of personal protective equipment (PPE) vary widely.

During the outbreaks of both SARS-CoV and MERS-CoV, patient-to-patient and patient-to-HCP transmission occurred in healthcare settings.^3,4^ Although the level of risk of transmission across hospital occupants (to HCPs and others) falls on a spectrum, all of these groups pose unique challenges when it comes to reducing transmission. In hospital settings, performing aerosol-generating procedures (AGPs, eg intubation, suction, bronchoscopy, cardiopulmonary resuscitation) or using a nebulizer on a SARS patient facilitated patient-to-HCP transmission.^3,5,6^ Overcrowding in emergency rooms, poor compliance with IPC measures, and contamination of the environment also contribute to viral spread.^7–11^

In healthcare settings, the most common pathway of human-to-human transmission has been the contact of the mucosae with infectious respiratory droplets or fomites.^12^ However, prior studies have also detected coronaviruses in sputum, nasal or nasopharyngeal secretions, endotracheal aspirate, bronchoalveolar lavage, urine, feces, tears, conjunctival secretions, and blood and lung tissues.^13–16^ Other research has also shown that SARS-CoV can survive in sputum, serum, and feces for at least 96 hours and in urine for 72 hours,^17^ and it can survive on surfaces up to 9 days.^18^ Thus, the recommended mitigation strategies may need to be sufficiently broad to control these transmission modes.

The COVID-19 IPC guidelines have been adopted and or developed based on the knowledge gained from experience during responding MERS-CoV or SARS-CoV outbreaks.^19–22^ However, the available published literature to date have indicated that SARS-CoV-2 is genetically similar to, but distinct from, SARS-CoV^22–24^ in terms of transmissibility, viral shedding, and other characteristics.^25–28^ Therefore, a critical review of the available literature related to the COVID-19 outbreak is essential as part of informing and updating IPC guidelines. In this study, we examined the current recommendations for IPC in light of what is known to date about COVID-19.

## Methods

We reviewed global COVID-19 IPC guidelines from the World Health Organization (WHO), the US Centers for Disease Control and Prevention (CDC), and the European Centre for Disease Prevention and Control (ECDC). We selected these international guidelines because they are commonly used as a reference globally.^29,30^ Guidelines from 2 high-income countries (Australia and the United Kingdom) and 1 middle-income country were also selected. We searched publications in English on ‘PubMed’ and Google Scholar for the period between January 1 and April 27, 2020, using the following search terms: “2019-nCoV” or “COVID-19” or “2019 novel coronavirus” or “SARS-CoV-2.” To identify COVID-19 IPC guidelines, we visited the websites of the international public health agencies such as CDC, ECDC, WHO, as well as the Australian Government Department of Health, the Bureau of Disease Prevention and Control of the National Health Commission of the People’s Republic of China, and Public Health England. Using the aforementioned terms, we also undertook a Google search for newspaper articles, reports, and updates related to the disease.

### Data management and analysis

We extracted information related to COVID-19 transmission dynamics, clinical presentations, and exposures that may facilitate the transmission while reviewing the literature. For guidelines, we extracted title, country or organization, department, target audience, and the different control measures recommended to control COVID-19. The lead author extracted the information from the guidelines, and all coauthors reviewed and validated it. We performed a content analysis of all data and summarized it under certain themes, and we then compared and contrasted our findings as they related to COVID-19 IPC measures.^31^

## Results

### Transmission dynamics

The SARS-CoV-2 is a zoonotic virus, and bats are assumed to be the reservoir.^23,32^ The suspected mode of COVID-19 transmission in Wuhan is from bats to humans; this animal served as an intermediate host that facilitated the transfer of this virus to humans.^23^ SARS-CoV-2 can be spread via droplets and aerosols (in a closed environment with high concentration of aerosols) transmitted from human to human through everyday interactions and by contact (eg, a person touches the patient or object contaminated with the virus).^21,22,33–43^ van Doremalen et al^44^ found that SARS-CoV-2 may remain viable in aerosols for up to 3 hours and on surfaces for up to several days.^44,45^ Public Health England classified COVID-19 as an airborne, high-consequence, infectious disease in the United Kingdom.^21^ Transmission may occur presymptomatically, during the incubation period, or even after recovery.^46,48^ Like influenza and other respiratory pathogens, SARS-CoV-2 may also be transmitted through respiratory droplets through coughing and sneezing.^49^ The CDC team reasoned that when an infected person coughs or sneezes, the large respiratory droplets expressed from the patients’ mouth and nose are likely to transmit the virus from the infected patient to a healthy person.^50^ The propelled droplets can land directly on the mucous membrane of the mouth, nose, or eyes of a nearby person or on the surface of objects.^49^ These droplets may travel up to ~4 m^51^ and may increase the risk of infection to HCPs.^52^ Guo et al^51^ also identified SARS-CoV-2 on shoe soles of HCPs working in intensive care units (ICUs); therefore, shoes can carry the virus. In an experimental study conducted by van Doremalen et al,^44^ SARS-CoV-2 remained viable on plastic and stainless-steel surfaces for up to 3 days. Moreover, SARS-CoV-2 RNA was identified on a cruise ship 17 days after the ship was vacated.^45^ AGPs, such as bronchoscopy, bronchial suction, tracheal intubation, and sputum induction, may generate aerosols containing the virus and increase the risk of transmission.^19,42^ These modes of transmission may contribute to spreading the virus in healthcare settings, including super-spreading events,^53^ and they inform guidance for IPC in healthcare settings.

### Exposures that may facilitate risk of infection

The incubation period of COVID-19 is 2–14 days.^64^ Backer et al^65^ estimated the mean incubation period to be 6.4 days (95% confidence interval [CI], 5.6–7.7). The available findings showed that transmission of SARS-CoV-2 may occur before and after symptom onset.^45^ Zou et al^27^ found modest viral loads on nasal and throat swabs early in the illness, with viral loads peaking ~5 days after symptom onset. The virus can be detected until 15 days from onset of illness and can be transmitted throughout the illness episode.^27^ Sharing a toilet in healthcare settings can also be a source of infection; the SARS-CoV-2 has been detected in toilet bowls and sinks.^66,67^ Inappropriate selection of PPE may also put HCPs at risk of infection.^68^ Exposure to AGPs was identified as a risk factor for acquiring COVID-19,^42^ but the others drivers of transmission and the exact mode of transmission remain uncertain. For example, blood, saliva, and stool, of COVID-19 patients have been tested positive for SARS-CoV-2,^60,63,69^ but the precise role these body fluids play in disease transmission in healthcare settings and the ways in which they may be transferred remain uncertain.

### Occupational risk

As of April 8, 2020, >22,000 HCPs have been infected with COVID-19 in 55 countries.^70^ HCPs comprise ~11% of all reported COVID-19 cases in Italy,^70^ 13.6% in Spain,^71^ ~14% in the United Kingdom,^72,73^ and 3.8% in China.^70^ One of the largest known outbreaks of hospital-acquired COVID-19 was reported in China among 17 (12.3%) of 138 patients and 40 (29%) of 138 HCPs in 1 hospital.^54^ Of the infected HCPs, 77.5% worked in general wards, 17.5% worked in the emergency department, and 5% worked in intensive care units.^54^ Li et al^74^ reported that no cases of COVID-19 occurred in HCPs before January 1, 2020.^74^ From January 1 to 11, 7 (3%) of 248 HCPs were infected, and from January 12 to 22, 7% (8/122) HCPs were infected, showing that healthcare-associated infections were increasing.^74^ A more recent study in a hospital in the United Kingdom showed ongoing transmission of COVID-19 among HCPs.^75^

### COVID-19 infection prevention and control guidelines

The Department of Health, Australia, the Bureau of Disease Prevention and Control of the National Health Commission of the People’s Republic of China, the CDC, the ECDC, Public Health England, and the WHO have published COVID-19 IPC guidelines that have targeted health administrators, HCPs, or public health units to implement IPC measures.^22,78–82^ Currently, the following IPC measures are in practice: suspected source control, use of personal protective equipment, rapid diagnosis, physical distancing, isolation, investigation, and follow-up of close contacts.^54^ All guidelines include administrative control, environmental control, and PPE, and the guidelines of Australia, the WHO, and the CDC also include engineering control. A comparison of the recommendations made in the guidelines is presented in Tables 1–3.

**Table 1.** Basic Infection Prevention and Control Measures Recommended in All International and National COVID-19 Guidelines

	Issuing Organization or Country					
	US Centers for Disease Control and Prevention	World Health Organization	European Centers for Disease Control and Prevention	The Department of Health, Australia	Bureau of Disease Prevention and Control, China	Public Health England
Title	Interim Infection Prevention and Control Recommendations for Patients with Confirmed 2019 Novel Coronavirus (2019-nCoV) or Persons Under Investigation for 2019-nCoV in Healthcare Settings^81^	Infection Prevention and Control During Health Care When Novel Coronavirus (nCoV) Infection Is Suspected^78^	Infection Prevention and Control for the Care of Patients With 2019-nCoV in Healthcare Settings^80^	CDNA National Guidelines for Public Health Units: Novel Coronavirus 2019 (2019-nCoV)^79^	Prevention and Control Guidelines on Novel Corona Virus Pneumonia, 5^th^ ed. [in Chinese]^22^	Guidance on Infection Prevention and Control for COVID-19^82^
Target audience	Hospital administrators and HCPs	Hospital administrators and HCPs	Hospital administrators and HCPs	Public Health Unit	Hospital administrators, HCPs, and community members	HCPs
**Administrative controls**						
Risk assessment	✓	✓	✓	✓	✓	✓
Train and educate HCPs on IPC	✓	✓	✓	✓^a^	✓	✓
Patient transfer precaution	✓	✓	✓	✓	✓	✓
Source control	✓	✓	✓	✓	✓	✓
Provide surgical masks to patients	✓^b^	✓	✓	✓	✓	✓
Early diagnosis	✓	✓	✓	✓	✓	✓
Suspected case isolation	✓	✓	✓	✓	✓	✓
Use of dedicated or disposable medical equipment	✓	✓	✓	✓	✓	✓**
Patients should be placed in single rooms	✓	✓	✓	✓	✓	✓
Respiratory hygiene	✓	✓	✓	✓	✓	✓
Waste management	✓	✓	✓	✓	✓	✓
Visitor management	✓	✓	✓	✓	✓	✓
Established reporting system	✓	✓	✓	✓	✓	✓
Disposition of patients	✓	✓	✓	✓	✓	✓
Contact and droplet precaution for COVID-19 patients care	✓	✓	✓	✓	✓	✓
**Environmental control measures**						
Negative pressure isolation room for AGPs	✓	✓	✓	✓	✓	✓
Contact, droplet and airborne precaution for AGPs	✓	✓	✓	✓	✓	✓
Routine clean and disinfect surfaces	✓	✓	✓	✓	✓	✓
Waste management	✓	✓	✓	✓	✓	✓
**Personal protective equipment**						
N95 or equivalent respirators for AGPs	✓	✓	✓	✓	✓	✓
Surgical masks for HCPs	✓^c^	✓	✓	✓	✓	✓
Gloves for HCPs	✓	✓	✓	✓	✓	✓
Gown for HCPs	✓	✓	✓	✓	✓	✓
Face shield for HCPs	✓	✓	✓	✓	✓	✓
Goggles/visor	✓	✓	✓	✓	✓	✓
Alcohol-based hand sanitizer for HCWs	✓	✓	✓^d^	✓	✓	✓
Hand wash with soap and water	✓	✓	✓	✓	✓	✓
Precaution during donning and doffing	✓	✓	✓	✓	✓	✓

Note. COVID-19, novel coronavirus 2019; HCPs, healthcare providers; CDNA, Communicable Disease Network Australia; IPC, infection prevention and control; AGP, aerosol-generating procedure; ICU, intensive care unit.

a Training for ICU staff.

b Depends on area of care and risk assessment.

c Only if N95 respirators are not available.

d If available.

**Table 2.** Discordance in Extended Administrative Infection Prevention and Control Measure Recommended in International and National COVID-2019 Guidelines

Issuing Organization or Country	CDC	WHO	ECDC	DHA	BDPCC	PHE
**Administrative controls**						
COVID-19 preparedness and response committee			✓	✓^c^	✓	
Plan for surge capacity			✓	✓^c^		✓
IPC focal point/group		✓			✓	
Spatial separation between patients	✓^a^	✓^b^		✓^c^		
Separate area for respiratory/suspected COVID-19 patients			✓	✓^c^		
Install physical barriers at hospital reception	✓					✓
Rapid triage	✓	✓		✓		
Triage outside facility	✓					
Cough etiquette	✓	✓		✓		
Separate toilet for patient	✓		✓			✓
Assess/ensure onsite availability of PPE			✓			✓
Provide hand and respiratory hygiene and cough etiquette supplies	✓	✓	✓			✓
Place known or suspected patient in AIIR/negative pressure room			✓	✓^d^	✓^d^	✓^d^
Cohorting confirmed patients in a ward with dedicated staff	✓^e^	✓^e^	✓^e^	✓	✓^e^	✓^e^
Decontaminate equipment if needed to share/reuse	✓	✓		✓	✓	✓
Visitor keep a distance of at least 1 m from a patient			✓			
HCPs wash hands after doffing			✓	✓		
Incinerating/sterilize clothing/bedding/utensils				✓	✓	✓
Monitor and manage ill and exposed HCPs	✓		✓	✓	✓	
HCP training on use of PPE	✓			✓^f^	✓	✓
Establish surveillance		✓		✓	✓	
Monitoring IPC compliance		✓			✓	
Patient education		✓		✓	✓	✓
Family caregiver/visitor education		✓				✓
Risk communication					✓	
Cleaning and disinfecting medical equipment	✓	✓			✓	✓
Maintain a register for visitor and follow-up for 14 days		✓	✓			
Post signs in public areas reminding symptomatic patients to remain 2 m away from HCPs	✓	✓		✓		✓
Disposal of PPE	✓			✓	✓	✓

Note. CDC, US Centers for Disease Control and Prevention; WHO, World Health Organization; ECDC, European Centers for Disease Control and Prevention; DHA, Department of Health, Australia; BDPCC, Bureau of Disease Prevention and Control, China; PHE, Public Health England; ICP, infection control and prevention; AIIR, airborne infection isolation room; HCPs, healthcare providers; PPE, personal protection equipment; ICU, intensive care unit.

a 1-meter distance between patients.

b 2-meter (6 ft) distance between patients.

c Included in state-level policies.

d Depends on availability.

e If single room is not available, patients are recommended to share a large room.

f Training for ICU staff.

**Table 3.** Discordance in Extended Environmental and Personal Protective Equipment Infection Prevention and Control Measure Recommended in International and National COVID-2019 Guidelines

Variable	Guideline Issuing Organization					
	US CDC	WHO	ECDC	DHA	BDPCC	PHE
**Environmental control measures**						
Staff engaged in environmental cleaning and waste management should follow contact and droplet precautions			✓	✓	✓	
Ensure adequate ventilation	✓	✓			✓	✓
Air disinfection					✓	
Cleaning and disinfecting medical equipment	✓	✓		✓	✓	✓
Disinfecting septic tanks					✓	
Pressure steam sterilization					✓	
Incinerating/sterilize clothing/bedding/utensils		✓		✓	✓	✓
Cleaning and disinfection electronic equipment			✓	✓^a^		
Decontamination of transport means/patient trolley			✓		✓	✓
**Personal protective equipment**						
Cloth masks for HCPs	✓^b^				✓	
Reuse use of PPE	✓	✓	✓	✓		✓
Cough etiquette	✓	✓		✓		
Decontaminate equipment if needed to share/reuse	✓	✓		✓	✓	✓
Use of PPE during patients transfer		✓		✓	✓	✓
Patient wear surgical mask during transfer		✓		✓		
N95 or equivalent respirators for routine care	✓^c^		✓^c^		✓	✓^d^
N95 fit testing	✓		✓	✓	✓	✓
N95 seal check	✓	✓		✓		
HCPs should wear scrubs			✓			✓
Injection safety practices		✓				
Use clean cloth towels			✓^e^			
Use cloth masks for patients	✓					
Family caregivers/visitors should use PPE	✓	✓	✓			✓
Visitor wear a surgical mask		✓	✓			
Visitor wear a cloth mask	✓					
Visitor wear gloves		✓	✓			
Visitor wear visor/goggles		✓	✓			
Visitor wear gown			✓			
PPE for social workers						✓
Dead body disposal standard precaution	✓	✓	✓	✓^a^	✓	✓
Use of corpse bags				✓^a^	✓	✓
Use of PPE while handling deceased body	✓	✓	✓	✓^a^	✓	✓
Use of PPE during postmortem procedure	✓			✓^a^	✓	✓
Contact and airborne precaution during postmortem autopsy	✓	✓	✓	✓^a^		✓
Autopsies should be performed in airborne infection isolation room	✓					✓

Note. CDC, US Centers for Disease Control and Prevention; WHO, World Health Organization; ECDC, European Centers for Disease Control and Prevention; DHA, Department of Health, Australia; BDPCC, Bureau of Disease Prevention and Control, China; PHE, Public Health England; HCPs, healthcare providers; PPE, personal protection equipment; ICU, intensive care unit.

a Included in state-level guidelines.

b When facemasks and N95 respirators are altogether unavailable.

c If available.

d Only in higher risk acute inpatient care.

e If paper towels are not available; included in a separate or state-level policy.

### Administrative controls

All guidelines recommend early diagnosis and isolation of COVID-19 patients in a single room, if available. In settings where single-room isolation facilities are limited, all of the guidelines recommend cohorting or group zoning of suspected COVID-19 patients in a well-ventilated room. The guidelines prioritize source control and recommend providing face masks to patients. The guidelines also recommend training for all HCPs regarding IPC measures. However, there are discrepancies in the guidelines regarding IPC measures. For example, the WHO recommends at least 1 meter distance between patients or between patients and HCPs when patients are cohorted in a large room, whereas Australia recommends 1.5 m of distance and the CDC recommends ~2 m (~6 ft) between patients. Moreover, 4 guidelines recommend patient education, and 3 guidelines suggest establishing surveillance in the hospital to monitor cross infection in patients and HCPs.

All of the guidelines highlight visitor controls in the hospitals. However, only China and the WHO discuss family members giving care in healthcare settings; they recommend that family caregivers use contact and droplet precautions while attending family members in the hospital. In addition, the ECDC guidelines recommend PPE for social workers when they provide care in healthcare settings.

### Environmental controls

All of the guidelines recommend that AGPs must be prioritized in a negative-pressure isolation room or in a well-ventilated room and that contact and airborne precautions should be followed during the AGP. To reduce room contamination in hospital settings, all of the guidelines recommend routine cleaning and disinfection of surfaces using disinfectants. The Chinese guideline also recommends air disinfectants using an air sterilizer and pressure steam sterilization. Incinerating or sterilizing patients’ clothing, bedding, and utensils are included in the guidelines from Australia, China, and the United Kingdom. Although the fecal–oral route of COVID-19 transmission has not yet been confirmed, the Chinese guidelines recommend disinfecting septic tanks. The CDC, ECDC, and UK guidelines recommended separate toilets for each patient. Although all of the guidelines recommend precautions during patient transfer, only the Chinese, ECDC, and UK guidelines emphasize decontaminating transportation means and trollies used by confirmed COVID-19 patients.

### Use of personal protective equipment

Due to the global supply shortages of PPE, almost all of the guidelines revised their initial recommendations related to PPE use. Of the 6 guidelines, 5 now recommend reuse of PPE following the manufacturers’ instructions. Considering the global scarcity of PPE supplies, the WHO, CDC, ECDC, Australian, and UK updated guidelines recommend surgical masks as an acceptable alternative to N95 respirators for HCPs during routine care, and N95 or equivalent respirators have been prioritized during AGPs. However, the recommendations around the type of face mask vary; for example, some guidelines recommend fluid-repellent surgical face masks, whereas others recommend general surgical masks.^82^ The CDC also recommends homemade cloth masks or homemade masks when a face mask is totally unavailable.^81^

As contact and droplet precautions, PPE measures, including wearing a surgical mask, and a gown, gloves, face shield, goggles and/or visors, and hand hygiene, have been recommended upon entering the patient’s room as well as removal of PPE upon leaving (Table 1). In all guidelines, alcohol-based hand sanitizers have been prioritized whenever available (Table 1). Fit testing and seal checks are an essential part of respirator use, but fit testing is recommended in 5 guidelines and a seal check is recommended in 3 guidelines. Precautions during donning and doffing are recommended in all guidelines. If an autopsy is required for a patient, the WHO, CDC, ECDC, and UK guidelines recommend the use of contact and airborne precautions during the autopsy. However, the WHO recommends performing autopsies in an adequately ventilated room, whereas the CDC recommends performing this procedure in airborne infection isolation room^85,86^

### Engineering control

Physical separation is efficient in reducing transmission of respiratory virus in hospital settings. The Australian, CDC, and WHO guidelines emphasize engineering control as an IPC measure. These guidelines recommend the following engineering control measures: spatial barriers or partitions to manage patients in triage areas, curtains around each bed in inpatient wards, closed suctioning systems for airway suctioning in intubated patients, and airflow management. The CDC guidelines also recommend installing physical barriers using glass or plastic windows in the hospital reception area.

### Corpse handling and management

All of the guidelines recommend standard precautions while handling dead bodies. Only the Australian, Chinese, and UK guidelines recommend the use of body bags. The Chinese guideline recommends putting cotton balls or gauze in the mouth, nose, ears, and anus, as well as any tracheotomy or open wound of the deceased body.^22^ All of the guidelines also state that a burial ritual may be allowed with standard precautions. A dedicated vehicle is recommended for postmortem transport.

## Discussion

In this review, we identified the transmission model and risk exposures of the COVID-19 pandemic. The identified signs and symptoms of the case patients suggest that SARS-CoV-2 can be transmitted through cough, sneeze, saliva, nasal secretion, stool, and vomit via droplet, aerosol, fecal–oral, or fecal–droplet transmission.^42,69^ However, currently discrepancies exist among the guidelines; not all documents acknowledge the 3 routes of transmission. To reduce exposures to SARS-CoV-2, all of the guidelines recommend early diagnosis and rapid isolation of COVID-19 patients. However, studies to date have indicated that rapid diagnosis of patients is challenging^87^ because the signs and symptoms of COVID-19 are nonspecific and may be confused with all microbial causes of respiratory tract infection.^87^ The nonspecific nature of the virus, as well as asymptomatic patients, may affect the IPC measures.

The recommendations regarding spatial separation between patients or between patients and HCPs are inadequate for droplet precautions in hospital settings. The recommendation of physical distance in the guidelines varies between 1 m and 2 m; however, a recent study has reported that the SARS-CoV-2 may travel >4 m.^51^ Moreover, environmental factors, such as air flow, humidity, and use of air conditioners or air mixing fans, may also influence the horizontal travel of droplets. An outbreak of COVID-19 linked to air conditioning has been reported in China.^88^ These reports indicate that revision of the spatial separation recommendation is warranted.

Although evidence that SARS-CoV-2 can be airborne is very limited, all of the guidelines recommend placing patients in a single room, if available. The exponentially large number of patients in several countries made the implementation of this isolation recommendation impossible due to the shortages of single isolation rooms.^89,90^ Therefore, cohorting patients in large shared rooms has become a practical alternative that is recommended in most updated guidelines. All of the international guidelines should make specific recommendations for hospitals that treat several patients in a large shared room. In addition, bed sheets and bed rails can be an important source of droplet and fomite transmission.^18^ None of the guidelines provided proper instruction on how to handle the bedding and clothing of COVID-19 patients. Because SARS-CoV-2 may remain viable on surfaces for days, a recommendation is needed for safe handling these items.

The presence of virus in stool samples indicates that the virus may also be transmitted through fecal–oral or fecal–droplet routes.^63,67,69^ Prior evidence of SARS coronavirus transmission through feces supports the likelihood of COVID-19 transmission via an oral–fecal or fecal–droplet route.^91^ In recent studies, investigators have detected SARS-CoV-2 in toilet bowls, sinks, and air.^66,92^ Toilet flushing may generate bioaerosols contaminated with pathogens. One study detected pathogenic microorganisms in air samples collected from hospital toilets, and the pathogen may remain viable in the air for at least 30 minutes after flushing suggest the possibility of fecal–droplet transmission.^93^ Specific recommendations are needed regarding the prevention of fecal–oral or fecal–droplet transmission in hospital settings.

Shortages of PPE are expected during pandemics due to high demand, and they have occurred in past epidemics as well.^94^ Due to the shortage of PPE, all guidelines recommend that HCPs should wear surgical mask as a droplet precaution and during specimen collection.^19,22^ The use of N95 or equivalent respirators is recommended only during AGPs in all guidelines.^19^ The virus may be transmitted through aerosols,^42,92^ and it can remain viable in aerosols for several hours.^44,92^ Therefore, face masks may not provide sufficient protection to HCPs due to their long and repeated exposure in confined spaces.^77^ In addition, the transmission dynamics of COVID-19 seems more like that of influenza than SARS-CoV.^27^ A randomized control study among HCPs exposed to influenza patients found that surgical masks may provide some protection to the wearers, probably by minimizing the frequency of times a person touches their nose and mouth^95^; however, surgical masks may not provide fully effective protection from respiratory pathogens because of leakage due to the loose fit of surgical masks.^96^ Considering the shortage of HCPs globally,^97^ the international guidelines should recommend optimal protection and IPC standards to protect frontline HCPs. Already, >22,000 HCPs have been infected, and many countries have reported ongoing nosocomial transmission of SAR-CoV-2 among HCPs.^70,72,98,99^ The role of face masks in protecting HCPs from SAR-CoV-2 has been questioned.^100^ We understand that a global shortage of N95 or equivalent respirators might have prompted the WHO, the UK, the ECDC, Australia, and the CDC to loosen their recommendations regarding face protection, but frontline HCPs should not be put at risk of infection. The face mask recommendation should be changed to N95 or equivalent respirators for all HCPs in all guidelines.

The guidelines should include a strong statement against the use of cloth or material masks, and HCPs should be encouraged not to wear 2 products simultaneously. Although 4 guidelines recommend the reuse of PPE or extended wear, no current guidelines address this behavior, and strict hand hygiene and donning/doffing procedures should be followed. For example, the UK guideline recommends that PPE be used between 2 and 6 hours, whereas the ECDC guidelines recommend wearing PPE for up to 4–6 hours.^80–82^ If countries resort to these strategies, it would be useful for the wider international community that observations studies be undertaken so that the results can be applied to future guidelines. Lastly, the WHO guidelines lack a recommendation on fit testing. It cannot be assumed that staff members have been fit tested for their respirators, so hospitals should be encouraged to fit test or at least fit check members of staff, including ancillary staff (ie, cleaning and support staff) and pharmacists who frequent the wards.

The recommendations should be updated regarding the disposition of patients after recovery and the use of standard precautions. Although all the guidelines make specific recommendations on this topic, some of the recommendations do not match our findings. For example, the WHO guideline recommends continuing standard precautions until a patient is asymptomatic. However, one study identified prolonged shedding of SARS-CoV-2 after recovery,^46^ and, therefore, special attention must be given to changing this recommendation. The discord in the recommendations on corpse handling may result in an increase in the risk of infection among the exposed. Corpse-to-human transmission of Ebola and Nipah viruses has been documented,^101,102^ and MERS-CoV was detected in the nasal secretions of a deceased human.^103^ SARS-CoV-2 has been detected in respiratory secretions, saliva and stool, and the virus may remain active in secretions and excreta from deceased bodies at least a few hours after death.^27,74,104–108^ Direct physical contact with bodies infected with the virus may increase the risk of infection. All of the guidelines should include recommendations on how to handle corpses and their management in hospitals.

The increasing numbers of COVID-19 cases among HCPs along with evidence of ongoing transmission in some hospitals suggest some that gaps in IPC measures should be revisited in the guidelines. Low- and middle-income countries often adopt international IPC guidelines as they stand or with modifications for the local context. Therefore, we recommend international guidelines consider the global context while recommending IPC measures.

In conclusion, SARS-CoV-2 may spread faster than the previous SARS-CoV. IPC measures should consider SARS-CoV-2 to spread as a droplet, an aerosol, and through the oral–fecal route. All of the guidelines should target these modes of transmission while recommending control measures. Because no drug or vaccine is publicly available for SARS-CoV-2, HCPs and other frontline outbreak responders must rely on IPC measures for safety. In addition, gaps always occur between the development of IPC guidelines, their introduction to target audience, and their implementation. During a public health emergency, international agencies may use an online platform to introduce IPC guidelines to HCPs in a shorter time. National authorities should provide training on the IPC guidelines to people at risk of infection.
